# Maternal green tea extract intake during lactation attenuates hepatic lipid accumulation in adult male rats exposed to a continuous high-fat diet from the foetal period

**DOI:** 10.29219/fnr.v64.5231

**Published:** 2020-10-05

**Authors:** Shojiro Yamasaki, Goh Kimura, Kazunari Koizumi, Ning Dai, Rahel Mesfin Ketema, Tomomi Tomihara, Yukako Ueno, Yuki Ohno, Shin Sato, Masaaki Kurasaki, Toshiyuki Hosokawa, Takeshi Saito

**Affiliations:** 1 Graduate School of Health Sciences, Hokkaido University, Sapporo, Japan; 2 Department of Nutrition, Aomori University of Health and Welfare, Aomori, Japan; 3 Faculty of Environmental Earth Science, Hokkaido University, Sapporo, Japan; 4 Institute for the Advancement of Higher Education, Hokkaido University, Sapporo, Japan; 5 Faculty of Health Sciences, Hokkaido University, Sapporo, Japan

**Keywords:** maternal supplements, high-fat diet, green tea extract, adult offspring, hepatic fat accumulation

## Abstract

**Background:**

Maternal lipid intake in the early postnatal period has a long-term effect on the possibility of fatty liver formation in children; besides, the importance of lipid consumption during lactation for children’s health has been suggested. Green tea extract (GTE) contains abundant catechins, and it has been reported to improve lipid metabolism and prevent fatty liver.

**Objective:**

The aim of this study was to examine the effects of maternal GTE intake during lactation on hepatic lipid accumulation in adult male rats exposed to a continuous high-fat (HF) diet from the foetal period.

**Methods:**

Pregnant Wistar rats received diets containing 13% (control-fat, CON) or 45% (high-fat, HF) fat. CON-fed mothers received the same diet during lactation, whereas HF-fed mothers received either HF diet alone or HF diet supplemented with 0.24% GTE. At weaning, male offspring were divided into three groups, i.e. CON/CON/CON, HF/HF/HF (HF-offspring) or HF/HF+GTE/HF (GTE-offspring), and were fed until 51 weeks.

**Results:**

A significant hepatic triglyceride (Tg) accumulation was observed in the HF-offspring when compared with the other offspring. This is presumed to be caused by the promotion of Tg synthesis derived from exogenous fatty acid due to a significant increase in diacylglycerol O-acyltransferase 1 and a decrease in Tg expenditure caused by decreasing microsomal triglyceride transfer protein (MTTP) and long-chain acyl-CoA dehydrogenase. On the other hand, attenuated hepatic Tg accumulation was observed in the GTE-offspring. The levels of the hepatic lipid metabolism-related enzymes were improved to the same level as the CON-offspring, and particularly, MTTP was significantly increased as compared with the HF-offspring.

**Conclusion:**

This study indicates the potential protective effects of maternal GTE intake during lactation on HF diet-induced hepatic lipid accumulation in adult male rat offspring and the possible underlying mechanisms.

## Popular scientific summary

- This study confirmed the long-term protective effects of maternal green tea extract (GTE) intake during lactation on hepatic lipid accumulation in adult male rats exposed to a continuous high-fat (HF) diet from the foetal period to 51 weeks of age.
- Maternal GTE intake prevents continuous HF diet-induced hepatic lipid accumulation of adult offspring by avoiding abnormalities of hepatic lipid metabolism, reducing lipogenesis from exogenous fatty acid and recovering lipid expenditure.

In current society, fatty liver is one of the major health issues worldwide, and many plant extracts have been used as treatments for fatty liver in many studies.

Green tea is a popular beverage made from the dried leaves of *Camellia sinensis*. Green tea extract (GTE) contains abundant polyphenolic compounds, including catechin, epicatechin, gallocatechin, epicatechin-3-gallate, epigallocatechin and epigallocatechin-3-gallate (EGCG). Among these components, EGCG is the most abundant tea polyphenol (1). GTE and EGCG have been well studied owing to their various beneficial effects on human diseases, including obesity, diabetes, liver diseases, inflammatory diseases and cancer (2–6).

GTE and its components have various therapeutic effects on lipid metabolism in animals fed a high-fat (HF) diet. Previous studies have reported that GTE and EGCG downregulate the expression of hepatic fatty acid synthases, such as fatty acid synthase (FAS), acetyl-CoA carboxylase (ACC) and sterol regulatory element-binding protein 1 (SREBP-1), and triglyceride (Tg) synthase such as diacylglycerol O-acyltransferase 1 (DGAT-1) and diacylglycerol O-acyltransferase 2 (DGAT-2) (7–9). GTE has also been reported to have the effect of activating beta-oxidation in mitochondria and suppressing fatty acid uptake (10, 11).

It is already known that personal lifestyle plays an essential role in hepatic lipid accumulation. On the other hand, studies suggest that maternal HF diet intake can affect the risk of liver steatosis developing in children (12–16).

Maternal GTE intake is expected to have protective effects against maternal HF diet-induced fatty liver in children. However, several studies pointed a concern about the adverse effects of maternal GTE intake during pregnancy (17–19). Therefore, in this study, we examined long-term effects of maternal GTE intake during lactation. Previous studies indicated that maternal polyphenol intake during lactation period attenuated hepatic lipogenesis in adult rat offspring (20, 21). Some studies inferred that maternal HF diet intake during lactation strongly induces obesity in offspring than during pregnancy (16, 22). Additionally, maternal GTE intake during lactation showed no adverse effect on mothers and offspring in previous studies (23, 24).

Studies have demonstrated long-term protective effects of maternal GTE intake against diet-induced kidney diseases in rat offspring (23, 24). However, there are limited studies about the long-term effects of maternal GTE intake during the lactation period on potential hepatic lipid accumulation of HF diet-fed offspring.

This study was conducted to examine the effects of maternal GTE intake during the lactation period on hepatic lipid accumulation in adult male rat offspring exposed to maternal and post-weaning HF diet.

## Materials and methods

### Animal treatment

**Fig. 1:**
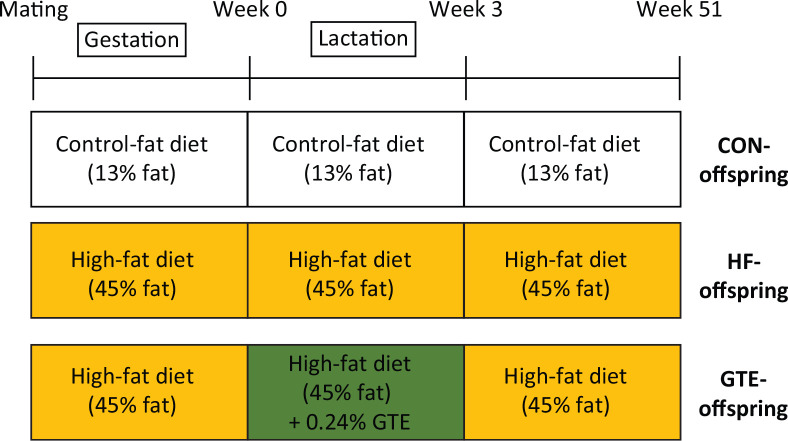
Experimental design. CON-offspring: a control-fat diet (13% fat) during gestation, lactation and after weaning; HF-offspring: a high-fat diet (45% fat) during gestation, lactation and after weaning; GTE-offspring: HF diet during gestation, 0.24% GTE-containing HF diet during lactation and HF diet after weaning.

The Animal Research Committee, Aomori University of Health and Welfare, approved this study, and all experimental procedures were performed following the Institutional Guidelines for Animal Experimentation. Seven-week-old, virgin, female Wistar rats obtained from CLEA Japan, Inc. (Tokyo, Japan), were maintained at a constant temperature of 23 ± 1°C under a 12:12 h light/dark cycle with ad libitum access to a commercial laboratory diet (MF diet; Oriental Yeast Co. Ltd, Tokyo, Japan) and tap water. At 12–13 weeks of age, we determined whether the female rats were in the appropriate oestrus cycle stage for mating using a vaginal impedance reader (Model MK-10C; Muromachi Kikai Co. Ltd, Osaka, Japan) routinely in the afternoon. A reading >3 kΩ indicated that the female rats were in pro-oestrus and presumably in oestrus. One appropriate female was mated with one male overnight. The presence of a vaginal plug the next morning indicated successful mating, and this day was noted as gestation day 0. As shown in Fig. 1, pregnant rats were randomly allocated to groups and fed control-fat diets (MF diet) (CON; *n* = 5) or HF diets (HF; *n* = 11) during gestation. The caloric content of the CON diet was 26% protein, 62% carbohydrate and 13% fat according to the manufacturer’s information. The caloric content of the HF diet was 16% protein, 39% carbohydrate and 45% fat. Following delivery, dams received either CON diet (CON/CON; *n* = 5) or HF diet (*n* = 11) during lactation. Those receiving the HF diet were further subdivided into receiving HF diet alone (HF/HF; *n* = 6) or HF diet containing 0.24% GTE (HF/HF+GTE; *n* = 5). The GTE, Polyphenon E, obtained from Mitsui-Norin Co. Ltd (Shizuoka, Japan) contained 80–98% total catechins by weight (the main component was EGCG, comprising ~65% of the material, as well as 0.4% caffeine). In this study, we administered 0.24% of GTE, which showed no adverse effect on mothers and offspring on lactation in previous studies (23, 24). At weaning (22 days of age), six male offspring from each group of dams were randomly selected and divided into the following three groups based on provided diet: CON/CON/CON (NF-offspring; *n* = 6), HF/HF/HF (HF-offspring; *n* = 6) and HF/HF+GTE/HF (GTE-offspring; *n* = 6). At 51 weeks of age, the male offspring were weighed, and blood samples were collected under anaesthesia after 12 h fasting. The livers and adipose tissues were removed immediately, rapidly rinsed with ice-cold saline and weighed. A portion of each liver was immediately frozen in liquid nitrogen and stored at −80°C before evaluation.

### Plasma lipids

Plasma samples were separated by centrifugation (800 × g for 10 min at 4°C) and tested for Tg and total cholesterol (TC) levels using commercially available kits (Wako Pure Chemical Industries Ltd, Osaka, Japan) according to the manufacturer’s instructions.

### Hepatic lipids

A liver sample weighing 100 mg was added to 450 μL of a solution of chloroform and ethanol (1:2, v/v) and homogenised. After incubation, 150 μL of chloroform was added to the mixture and blended. Next, 150 μL of distilled water was added and mixed again. The chloroform layer was separated from the samples after centrifugation (20,000 × g for 5 min at 25°C). Lipid fraction was obtained by the evaporation of chloroform. The obtained lipid was dissolved in isopropanol. Lipid levels were tested for Tg and TC using a commercially available kit (FUJIFILM Wako Pure Chemical Corporation, Osaka, Japan) according to the manufacturer’s instructions.

### Western blot analysis

For Western blot analysis, the liver samples were homogenised in a buffer on ice. The homogenate was centrifuged (20,000 × g for 20 min at 4°C), and the supernatant was collected. Next, the obtained supernatant was heated to avoid denaturation of protein and the protein concentration in the sample was measured by Bradford assay (25) using Protein Assay (BIO-RAD, Hercules, USA). Proteins in the sample were separated by SDS-PAGE, and biotinylated protein molecular weight markers (M&S TechnoSystems, Inc., Osaka, Japan) were used as protein standards. Proteins were then electrophoretically transferred onto a nitrocellulose membrane using the iBlot transfer system (Thermo Fisher Scientific K.K., Tokyo, Japan). The nitrocellulose membrane was incubated overnight at 4°C in a buffer containing 2% skim milk as a blocking solution. The membrane was then washed and exposed to primary antibodies, namely, ACC (3676; Cell Signaling TECHNOLOGY, Inc., Massachusetts, USA), DGAT-1 (GTX48577; Gene Tex, Inc., California, USA), DGAT-2 (NBP1-71701SS; Novus Biologicals, LLC, Colorado, USA), FAS (ab22759; Abcam, Tokyo, Japan), long-chain acyl-CoA dehydrogenase (LCAD; ab196655; Abcam, Tokyo, Japan), microsomal triglyceride transfer protein (MTTP; ab186446; Abcam, Tokyo, Japan), SREBP-1 (sc-13551; Santa Cruz Biotechnology, Inc., Texas, U.S.A.) and beta-actin (M177-3; Medical & Biological Laboratories Co., Ltd, Aichi, Japan), in the presence of a 1% blocking solution. Next, the membrane was again washed and exposed to secondary antibodies: anti-rabbit IgG IRDye 680 (926-68071; M&S TechnoSystems, Inc., Osaka, Japan) or anti-mouse IgG IRDye 800 (926-32210; M&S TechnoSystems, Inc., Osaka, Japan). Protein bands were quantified using Odyssey Infrared Imaging System (M&S TechnoSystems, Inc., Osaka, Japan), and protein levels were normalised against those of beta-actin from the same sample.

### Statistical analysis

Statistical analyses were performed using BellCurve for Excel (Social Survey Research Information Co., Ltd, Tokyo, Japan). Data were tested using one-way analysis of variance (ANOVA) followed by Fisher’s LSD test. Each value was expressed as mean ± SEM. In all cases, statistical significance was set at *P* < 0.05.

## Results

### Weight gain between week 3 and week 50

**Fig. 2:**
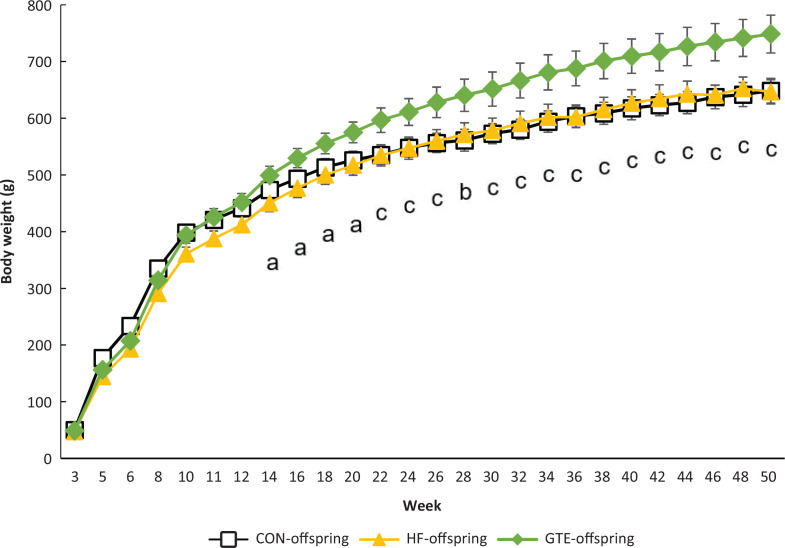
Weight gain of male rat offspring from week 3 to week 50. The open square (◻) indicates CON-offspring, the closed triangle (▲) indicates HF-offspring and the filled diamond (♦) indicates GTE-offspring. CON-offspring: a control-fat diet during gestation, lactation and after weaning; HF-offspring: a high-fat diet during gestation, lactation and after weaning; GTE-offspring: HF diet during gestation, 0.24% GTE-containing HF diet during lactation and HF diet after weaning. Values are expressed as mean ± SEM (*n* = 6). Data were tested using one-way ANOVA followed by Fisher’s LSD test. (a) *P* < 0.05 GTE-offspring versus HF-offspring; (b) *P* < 0.05 GTE-offspring versus CON-offspring; (c) *P* < 0.05 GTE-offspring versus both HF-offspring and CON-offspring.

Figure 2 shows the weight gain between week 3 and week 50 of the three offspring groups. No significant difference was found between HF-offspring and CON-offspring. GTE-offspring had higher body weights than CON-offspring after week 22 and HF-offspring after week 14.

### Morphological parameters at week 51

Table 1 shows morphological parameters at week 51. Significantly higher body weights were observed in GTE-offspring than in either of the other groups, whereas no significant difference was found between CON-offspring and HF-offspring. The liver of GTE-offspring was significantly heavier than HF-offspring. The kidney of HF-offspring was significantly lighter than the others. Epididymal fat of GTE-offspring was significantly heavier than CON-offspring. The perirenal fat in GTE-offspring was heavier than that of both CON- and HF-offspring. The relative liver and kidney weight of both HF-offspring and GTE-offspring was significantly lighter compared with that of CON-offspring. The relative epidydimal fat weight of only GTE-offspring was significantly heavier than that of CON-offspring. Although the relative perirenal fat weight of GTE-offspring was much heavier than HF-offspring, both of them were significantly heavier than CON-offspring.

**Table 1:** Morphological parameters of male rat offspring at week 51

Group	CON-offspring	HF-offspring	GTE-offspring
Body weight (week 3; g)	50.33 ± 2.72^a^	47.33 ± 2.85^a^	48.77 ± 3.07^a^
Body weight^†^ (week 51; g)	628.58 ± 23.03^a^	621.13 ± 11.0^a^	743.0 ± 29.64^b^
Liver (g)	15.08 ± 0.45^ab^	13.17 ± 0.38^a^	16.05 ± 0.97^b^
Kidney (g)	3.04 ± 0.06^b^	2.58 ± 0.07^a^	3.00 ± 0.11^b^
Epididymal fat (g)	9.20 ± 1.15^a^	12.04 ± 0.82^ab^	15.51 ± 2.19^b^
Perirenal fat (g)	24.79 ± 2.30^a^	37.86 ± 1.43^a^	60.39 ± 5.85^b^
Liver/BW (g/kg)	24.09 ± 0.84^b^	21.19 ± 0.38^a^	21.54 ± 0.58^a^
Kidney/BW (g/kg)	4.87 ± 0.18^b^	4.15 ± 0.08^a^	4.05 ± 0.06^a^
Epididymal fat/BW (g/kg)	14.54 ± 1.53^a^	19.37 ± 1.21^ab^	22.58 ± 1.62^b^
Perirenal fat/BW (g/kg)	39.23 ± 2.95^a^	60.99 ± 2.20^b^	80.44 ± 5.81^c^

CON-offspring: a control-fat diet during gestation and lactation, and after weaning; HF-offspring: a high-fat diet during gestation, lactation and after weaning; GTE-offspring: HF diet during gestation, 0.24% GTE-containing HF diet during lactation and HF diet after weaning. Values are expressed as mean ± SEM (*n* = 6). Data were tested using one-way ANOVA followed by Fisher’s LSD test. Values with the different superscript letters in the same line are statistically different (*P* < 0.05).

† At sacrifice.

### Triglyceride and total cholesterol concentrations in the liver and plasma

Table 2 shows lipid concentrations in the liver and plasma of offspring at 51 weeks of age. Significantly higher levels of hepatic Tg and lower levels of plasma Tg were observed in HF-offspring than in CON-offspring, whereas no significant differences were found between CON-offspring and GTE-offspring. A similar pattern of change was observed in hepatic and plasma TC levels, with a significant difference between CON-offspring and HF-offspring found in both sources.

**Table 2:** Lipid concentrations in the liver and plasma of offspring at week 51

Group	CON-offspring	HF-offspring	GTE-offspring
Liver Tg (mg/g wet tissue)	15.93 ± 2.66^a^	29.63 ± 6.22^b^	23.99 ± 2.73^ab^
Liver TC (mg/g wet tissue)	2.15 ± 0.17^a^	3.00 ± 0.37^b^	2.37 ± 0.14^ab^
Plasma Tg (mg/dL)	150.47 ± 9.36^b^	87.8 ± 10.38^a^	166.47 ± 23.73^b^
Plasma TC (mg/dL)	81.71 ± 4.83^b^	53.31 ± 1.93^a^	69.79 ± 5.36^ab^

Hepatic triglyceride (Tg) and total cholesterol (TC) content (mg/g wet tissue), and plasma Tg and TC level (mg/dL) in male rat offspring at week 51. CON-offspring: a control-fat diet during gestation, lactation and after weaning; HF-offspring: a high-fat diet during gestation, lactation and after weaning; GTE-offspring: HF diet during gestation, 0.24% GTE-containing HF diet during lactation and HF diet after weaning. Values are expressed as mean ± SEM (*n* = 6). Data were tested using one-way ANOVA followed by Fisher’s LSD test. Values with the different superscript letters in the same line are statistically different (*P* < 0.05).

### Changes in hepatic lipid metabolic enzymes and transcription factor

#### Fatty acid synthases

**Fig. 3:**
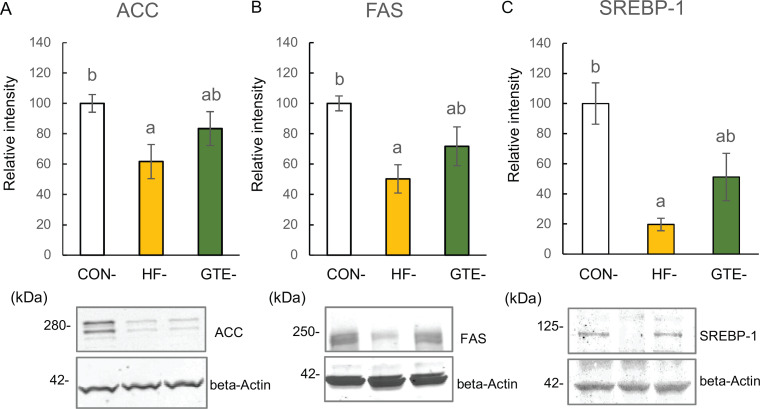
Protein expressions of acetyl-CoA carboxylase (ACC; A), fatty acid synthase (FAS; B) and sterol regulatory element-binding protein 1 (SREBP-1; C) in the liver of male rat offspring at week 51. For each condition, densitometric analysis was conducted relative to beta-actin. The left column indicates CON-offspring, the middle column indicates HF-offspring and the right column indicates GTE-offspring. CON-offspring: a control-fat diet during gestation, lactation and after weaning; HF-offspring: a high-fat diet during gestation, lactation and after weaning; GTE-offspring: HF diet during gestation, 0.24% GTE-containing HF diet during lactation and HF diet after weaning. Values are expressed as mean ± SEM (*n* = 6). Data were tested using one-way ANOVA followed by Fisher’s LSD test. The bars in the different serial shown by the different letters significantly differ from each other at a level of 5%.

Significantly lower levels of hepatic ACC (Fig. 3A) and FAS (Fig. 3B) were observed in HF-offspring than in CON-offspring. In GTE-offspring, levels of those enzymes were decreased but not significantly changed. To examine why these enzymes were reduced, hepatic SREBP-1 levels were measured. SREBP-1 is a transcription factor that contributes to lipogenesis and regulates expression of ACC and FAS (26). Significantly lower levels of SREBP-1 were observed in HF-offspring than in CON-offspring; this decrease was attenuated in GTE-offspring (Fig. 3C).

#### Triglyceride synthases

**Fig. 4:**
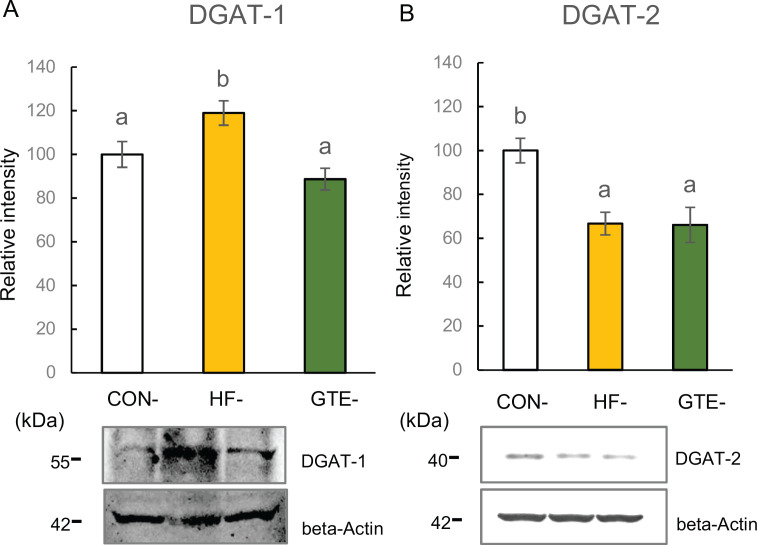
Protein expressions of diacylglycerol O-acyltransferase 1 (DGAT-1; A) and diacylglycerol O-acyltransferase 2 (DGAT-2; B) in the liver of male rat offspring at week 51. For each condition, densitometric analysis was conducted relative to beta-actin. The left column indicates CON-offspring, the middle column indicates HF-offspring and the right column indicates GTE-offspring. CON-offspring: a control-fat diet during gestation, lactation and after weaning; HF-offspring: a high-fat diet during gestation, lactation and after weaning; GTE-offspring: HF diet during gestation, 0.24% GTE-containing HF diet during lactation and HF diet after weaning. Values are expressed as mean ± SEM (*n* = 6). Data were tested using one-way ANOVA followed by Fisher’s LSD test. The bars in the different serial shown by the different letters significantly differ from each other at a level of 5%.

The level of hepatic DGAT-1 was significantly higher in HF-offspring than in CON-offspring and GTE-offspring, whereas no significant change was found between GTE-offspring and CON-offspring (Fig. 4A). Significantly lower DGAT-2 levels were observed in both HF-offspring and GTE-offspring than in CON-offspring (Fig. 4B).

#### Lipid expenditure: secretion and beta-oxidation

**Fig. 5:**
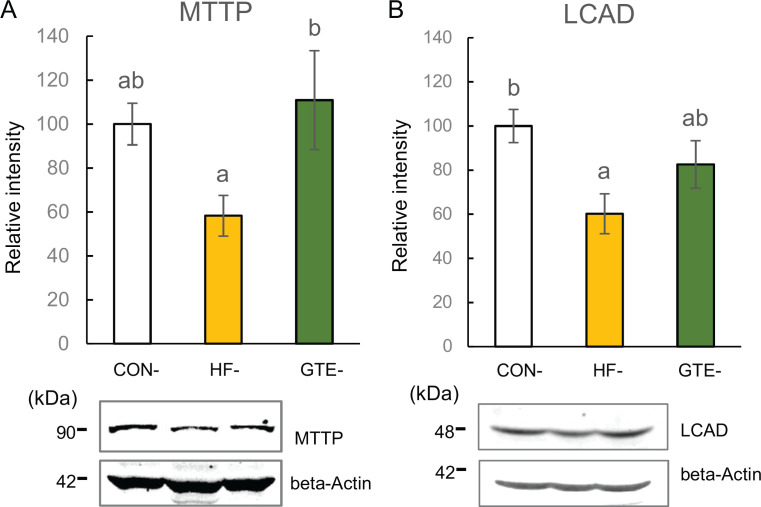
Protein expressions of microsomal triglyceride transfer protein (MTTP; A) and long-chain acyl-CoA dehydrogenase (LCAD; B) in the liver of male rat offspring at week 51. For each condition, densitometric analysis was conducted relative to beta-actin. The left column indicates CON-offspring, the middle column indicates HF-offspring and the right column indicates GTE-offspring. CON-offspring: a control-fat diet during gestation, lactation and after weaning; HF-offspring: a high-fat diet during gestation, lactation and after weaning; GTE-offspring: HF diet during gestation, 0.24% GTE-containing HF diet during lactation and HF diet after weaning. Values are expressed as mean ± SEM (*n* = 6). Data were tested using one-way ANOVA followed by Fisher’s LSD test. The bars in the different serial shown by the different letters significantly differ from each other at a level of 5%.

MTTP plays an important role in the association of lipids with apo-B protein to form very low-density lipoprotein (VLDL) and lipid secretion into the blood (27). In HF-offspring, hepatic MTTP level was downregulated but not significantly lower than CON-offspring (*P* = 0.0672), whereas in GTE-offspring, it significantly upregulated compared with HF-offspring, and no significant change was found between CON-offspring and GTE-offspring (Fig. 5A). LCAD plays an important role in mitochondrial fatty acid oxidation (28). A significantly lower hepatic LCAD level was observed in HF-offspring than in CON-offspring, whereas there was no significant change in GTE-offspring (Fig. 5B).

## Discussion

This study examined hepatic lipid metabolism in adult male rat offspring to investigate the effects of maternal GTE intake on hepatic lipid accumulation induced by maternal and post-weaning HF diet.

In HF-offspring livers, downregulated fatty acid synthesis, fatty acid oxidation and *de novo* Tg synthesis were observed, whereas exogenous Tg synthesis was upregulated. There were also indications that lipid secretion from the liver was attenuated.

ACC and FAS are widely known as crucial enzymes for *de novo* fatty acid synthesis from acetyl-CoA, and their expression is regulated by SREBP-1 (26). Our Western blot results indicate lower expression of both ACC and FAS, and their transcriptional regulator SREBP-1 suggests that hepatic fatty acid synthesis in HF-offspring was attenuated through a transcriptional mechanism. Both DGAT-1 and DGAT-2 mediate the binding of diacylglycerol and acyl-CoA at the final stage of Tg synthesis. However, each uses a different substrate, and it has been found that DGAT-1 uses exogenous substrates -- fatty acid derived from diet -- whereas DGAT-2 uses *de novo* fatty acid as its substrate (29).

In the liver of HF-offspring, *de novo* fatty acid synthesis is suppressed, whereas the exogenous fatty acid influx into the liver of HF-offspring is considered to be increased by HF diet intake (30). Thus, changes in the expression of the two Tg synthases can be related to changes in the amount of each substrate. Donnelly et al. (31) reported that about 60% of the lipids present in the liver were derived from exogenous fatty acids; this suggests that the increased DGAT-1 levels in HF-offspring demonstrated in this study contributed to the hepatic lipid accumulation seen in HF-offspring. It has also been reported that a mechanism exists to prevent excessive accumulation of lipids even if Tg synthesis is extraordinarily promoted. Yamazaki et al. (32) reported that the overexpression of DGAT-1 increased Tg synthesis, whereas hepatic lipid accumulation was not significant due to increased Tg secretion with VLDL. However, in this study, the level of hepatic MTTP, an enzyme that greatly contributes to lipid release with lipoprotein (27, 33), was decreased in HF-offspring. Lower plasma lipid in HF-offspring supports the idea that lipid secretion from HF-offspring was attenuated. Moreover, LCAD, a protein that contributes to mitochondrial fatty acid oxidation (28), was also decreased in HF-offspring. These results suggest that the mechanism that prevents excessive accumulation of lipids in the liver is not functioning correctly in the HF-offspring. It is speculated that lipid accumulated in the liver of HF-offspring due to a lipid metabolism disorder: excess promotion of Tg synthesis using exogenous fatty acid and attenuated lipid expenditure by secretion or oxidation.

The changes in levels of hepatic lipid metabolism-related proteins in the GTE-offspring showed a similar pattern as that seen in the HF-offspring in most proteins, but unlike the HF-offspring, the changes were not significant when compared with the CON-offspring. This indicates that maternal GTE intake ameliorated abnormalities in hepatic lipid metabolism in the offspring. Only the level of DGAT-2 was significantly lower in GTE-offspring than the CON-offspring. It is supposed to be related to that *de novo* fatty acid synthesis tended to be attenuated from the CON-offspring and that fatty acid oxidation was performed normally. Whereas the other proteins changed in a similar pattern to those in HF-offspring, the level of MTTP in GTE-offspring was significantly higher than that in HF-offspring. As HF diet intake reduces MTTP protein levels (34), this suggests that GTE intake during lactation modulates MTTP expression in the long term. Moreover, reports have shown an association between lipid secretion from the liver and hepatic lipid accumulation (32, 33). Thus, an increase in the hepatic MTTP level in the GTE-offspring suggests that the effect of maternal GTE intake on the hepatic lipid secretion system played a major role in suppressing hepatic lipid accumulation. This study revealed that maternal GTE intake suppressed HF diet-induced hepatic lipid accumulation in offspring by amelioration of abnormal lipid metabolism. It further suggests the importance of its effects on lipid secretion.

Interestingly, despite being continuously fed an HF diet, HF-offspring did not differ significantly from CON-offspring in body weight, unlike GTE-offspring. A possible reason is that the development of other organs and tissues such as liver and kidney was suppressed, whereas the relative weight of perirenal fat in HF-offspring was increased. The reduced relative liver and kidney weights of both HF-offspring and GTE-offspring inferred that continued exposure to the HF diet suppresses organ development. On the other hand, GTE-offspring accumulated much fat in adipose tissue and gained weight. Abnormal lipid metabolism observed in HF-offspring and improved lipid metabolism and suppressed lipid accumulation in the liver of GTE-offspring are considered to be closely related to lipid accumulation in adipose tissue and weight gain of offspring. However, further studies are needed to elucidate the underlying mechanisms and impacts on health.

One of the key findings in this study was that lipid metabolism in the HF- and GTE-offspring were significantly different at 51 weeks of age. In addition, from the change in weight gain from week 14 to week 51, it can be inferred that the changes in metabolism have been continuously persisted. This indicates long-term protective effects of short-term treatment for the mother during lactation on hepatic lipid accumulation in children. GTE is transferred to the child via breast milk, but EGCG, the main component of GTE, has been shown to be rapidly excreted and not retained (35, 36). Therefore, it is considered that the GTE itself, taken by the mother during the lactation period, did not remain in the offspring but affected the early life of offspring, and these effects were maintained until late adulthood. Epigenetic alteration is one of the possible mechanisms that may play a role in maintaining these effects from childhood to late adulthood. Previous studies have shown that the expression of some lipid metabolism-related proteins is under epigenetic regulation (34, 37, 38), which can be induced during early life by maternal nutrient status. Ehara et al. (37) reported that maternal HF diet intake reduces DNA methylation of glycerol-3-phosphate acyltransferase 1 promoter region in offspring and promotes Tg synthesis. Other studies (39, 40) also showed treatment for mothers modulates epigenetic regulation of lipid metabolism-related gene expression in offspring. Moreover, it has also been suggested that epigenetic alterations that occur during early life can be preserved until post-growth (41). From previous studies, it is also inferred that there are long-term effects of GTE intake on the expression of DNA methyltransferase 1 and 3a, which act in a compensatory manner against maternal malnutrition (24, 42). The hepatic lipid accumulation observed in HF-offspring may have been caused by maternal HF diet-induced epigenetic alterations in hepatic lipid metabolism. It has been suggested that maternal GTE intake during lactation has effects of improving hepatic lipid metabolism in offspring through epigenetic alterations. On the other hand, there are many possible factors that may contribute to hepatic lipid accumulation. It has been reported that insulin resistance and chronic inflammation contribute to hepatic lipid accumulation (43). There are also suggested effects of maternal HF diet and GTE intake on the formation of predispositions to hepatic lipid accumulation in offspring (44–46). Therefore, further study is necessary to clarify the mechanism underlying the preventive effects of maternal GTE intake on hepatic lipid accumulation.

## Conclusion

This study examined the effects of maternal GTE intake during lactation on hepatic lipid metabolism in adult male rat offspring exposed to a continuous HF diet from the foetal period to late adulthood. In male rat offspring exposed to maternal and post-weaning HF diet, lipid accumulation in the liver was induced by increased hepatic Tg synthesis and suppression of hepatic lipid expenditure. It was shown that GTE intake during lactation improved hepatic lipid metabolism, including lipid release, and suppressed lipid accumulation in the liver. It is suggested that maternal GTE intake during the lactation period affects offspring until late adulthood through epigenetic alterations. This study implies that maternal GTE intake during lactation is effective in protecting children from hepatic lipid accumulation, whereas further studies are needed to clarify the mechanism.
